# Child Morbidity and Disease Burden in Refugee Camps in Mainland Greece

**DOI:** 10.3390/children6030046

**Published:** 2019-03-17

**Authors:** Asterios Kampouras, Georgios Tzikos, Eustathios Partsanakis, Konstantinos Roukas, Stefanos Tsiamitros, Dimitrios Deligeorgakis, Elisavet Chorafa, Maria Schoina, Elias Iosifidis

**Affiliations:** 1 Pediatric Department, 424 General Military Hospital, Thessaloniki 56429, Greece; 2 Surgery Department, 424 General Military Hospital, Thessaloniki 56429, Greece; giorgos-t@hotmail.com; 3 Medical Service, Hellenic Army, Thessaloniki 56429, Greece; epartsa@gmail.com (E.P.); stevetsam@gmail.com (S.T.); d.deligeorgakis@gmail.com (D.D.); 4 Internal Medicine Department, 424 General Military Hospital, Thessaloniki 56429, Greece; rooky17@gmail.com; 5 School of Medicine, Aristotle University of Thessaloniki, Thessaloniki 54124, Greece; elsachorafa@hotmail.gr; 6 Nephrology Department, Hippokration General Hospital, Aristotle University of Thessaloniki, Thessaloniki 54642, Greece; maninaschoina@gmail.com; 7 Infection Control Committee, Hippokration General Hospital, Infectious Diseases Unit, 3rd Department of Pediatrics, Aristotle University of Thessaloniki, Thessaloniki 54642, Greece; iosifidish@gmail.com

**Keywords:** child morbidity, disease burden, refugee crisis

## Abstract

The crisis conflicts in Syria have forced a lot of people to relocate and live in mainland Greece, where they are hosted in refugee camps. In the present study, our aim was to assess child morbidity and overall disease burden in two camps in northern Greece during a six-month winter period. A primary health care office was founded in each camp. Refugees of all ages with health problems were examined daily by specialty doctors. Cases were classified into two categories: Infectious or non-infectious. In total, 2631 patients were examined during this period (out of the 3760 refugees hosted). Of these patients, 9.8% were infants, 12.7% were toddlers, and 13.4% were children. Most of the visits for children aged less than 12 years old were due to infectious diseases (80.8%). The most common sites of communicable diseases among children were the respiratory tract (66.8%), the skin (23.2%), and the urinary (3.2%) and gastrointestinal tracts (6.2%). Non-communicable diseases were mostly due to gastrointestinal (20.2%), respiratory (18.2%), surgical (13.1%), and allergic (10.3%) disorders. Infants, toddlers, and children suffered more frequently from respiratory infections, while in adolescents and adults, non-infectious diseases were more common. Toddlers and children were more likely to fall ill in comparison to infants. Conclusions: During the winter period, infectious diseases, especially of the respiratory tract, are the main reason for care seeking among refugees in Greek camps, with toddlers suffering more than other age groups. The overall mortality and referral percentage were low, indicating that adequate primary care is provided in this newly established refugee hosting model.

## 1. Introduction

The ongoing crisis with the intense conflicts in Syria and neighboring countries has forced more than 6 million people to abandon their home countries, seeking a safer place to relocate to [1]. The deteriorating situation in Syria accounts for the increased amount of refugees, whose primary destinations are countries in central and northern Europe, which they have tried to reach by following the eastern Mediterranean route through Turkey and Greece [1,2]. However, the closure of the Greek-Former Yugoslav Republic of Macedonia (FYROM) border on February 2016 has caused the popular “Balkan route” to shut down. Refugees and migrants, for whom Greece was mainly a transitional location, had their dream interrupted [3]. Adding to that, the implementation of the EU-Turkey agreement on March 2016 drastically limited the arrival of more refugees to Greece, who instead will likely try to reach Europe through irregular ways, risking their lives [2,3,4]. As a result, more than 62,000 refugees are currently stranded in Greece, with no prospect of moving to any northern countries [5]. 

Moreover, refugee camps (or “hotspots”), originally aimed for short-term stays, had to be turned into long-term shelters, and since they were not designed to host such large amounts of refugees, they became overcrowded. Therefore, new challenges have arisen as the facilities are not adequate, thus the living conditions are considered inappropriate. Another important issue that has come up is that these camps may host a considerable number of unaccompanied minors, especially children with an unknown vaccination status, suffering from the war and the uncertainty of the journey to a new place to resettle. Consequently, both their physical and mental health have been disturbed [6]. These facts raise questions about the burden of disease and the prevalence of vaccine-preventable diseases. Lately, there has been increased interest and a need for evidence-based reports on migrant health and its impact on the national health systems of European countries [7,8], along with a lack of adequate data on the health condition of the current refugee wave. 

Therefore, the aim of the present study was to: (a) assess the burden of disease in two refugee camps in mainland northern Greece during an autumn-winter period, and (b) to compare the burden of disease between different age groups, also checking child morbidity.

## 2. Methods

Our study was conducted in two refugee camps in northern Greece during the autumn-winter period from October 2016 to March 2017. These camps hosted about 250 refugees each, who had mostly travelled from Afghanistan and Syria. Of these refugees, about 280 were adults, and about 220 were under 18 years old. Refugees arrive at these camps a few days after entering the country, usually by sea. Upon entrance they are vaccinated, according to the National Immunization Schedule, and after asylum is granted to them they are relocated to camps in mainland Greece, in order to avoid congestion at entrance points. In refugee camps in mainland Greece, refugees stay for an average period of 1.5 months until they find housing in one of Greece’s cities. Most of the children who arrive are accompanied by family members. The status of previous vaccination was undefined in most cases. A primary health care office was founded in every camp and was ran under the supervision of the medical service of the Hellenic Army. Refugees with health problems were examined on a daily basis by specialty doctors and were referred to tertiary or university hospitals when further care was needed. In cases of emergency, patients were transferred to hospitals with the assistance of ambulances from the National Emergency Aid Center. Communicable diseases were categorized into four groups according to the site of the infection, which included respiratory, urinary, gastrointestinal, and skin infections. In cases of febrile illness, C-Reactive Protein (CRP) values were measured on site. Regarding non-communicable diseases, patients were also classified in categories according to the system that was affected (cardiovascular, surgical, obstetric, gastrointestinal, respiratory, allergic, orthopedic, hematological, endocrinological, or others). A database of refugees providing medical care was created with the following fields: Age, gender, reason for seeking medical advice, underlying condition, medical diagnosis, CRP, and referrals. Each patient was recorded once every time they sought medical advice in order to avoid duplicate recorded data. All patients were informed about the study and provided informed written consent. All procedures performed in this study involving human participants were in accordance with the ethical standards of the institutional or national research committee and with the 1964 Helsinki declaration and its later amendments or comparable ethical standards.

### Statistical Analysis

Statistical analysis was conducted with the help of the IBM SPSS statistics software (24th edition). Baseline characteristics were summarized using appropriate descriptive statistics. The statistical significance was set to 0.05. The chi-square test was used to efficiently determine whether there was any association between the presence of infection or any other pathological condition status and different age groups, due to the fact that the variables were categorical. Finally, logistic regression was used to assess if there were any significant associations between the type of infection or pathological condition and different age groups. 

## 3. Results

### 3.1. Demographics

During the period from October 2016 to March 2017, approximately 3760 refugees found shelter in the two camps of our study, with 1654 of them being children (approximately 334 were infants (which accounts for 8% of whole population), 648 were toddlers (17%), and 672 were children (18%) 6–12 years old). In total, 2631 patients were examined at the primary care office. About 9.8% of them were infants, whereas 12.7% were toddlers. Children aged 6–12 years old accounted for 13.4% of office visits, whereas adolescents (12–18 years old) accounted for 7.9%. The age group of adults who were examined consisted of 1453 patients (55.2%). (Table 1) Regarding their gender, 48.2% of the refugees seeking medical care were male and 51.8% were female. 

### 3.2. Care Seeking or Reason for Care Seeking

**Figure 1:**
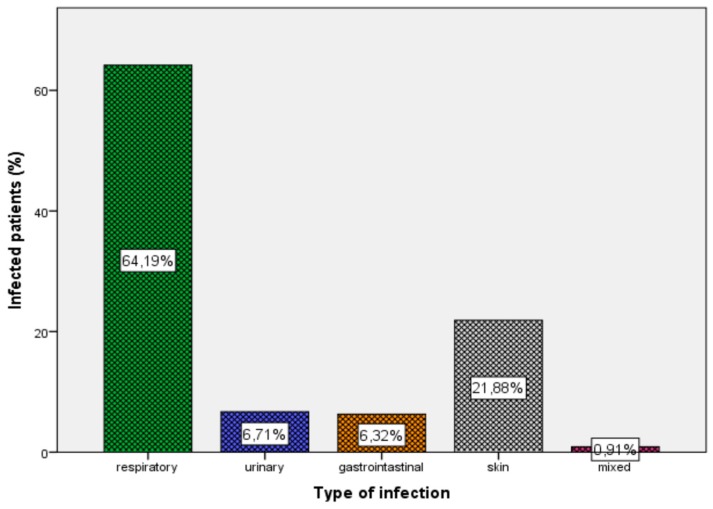
Type and percentage of infection in the whole population.

The most common reason for care seeking among the entire population was due to an infectious disease (58.4%). Regarding the rest of cases for care seeking, 41.6% were for non-communicable or non-infectious diseases. Regarding infectious diseases, the most common site of infection was the respiratory tract (64.2%), followed by the skin and soft tissue (21.9%), the urinary tract (6.7%), and the gastrointestinal tract (6.3%) (Figure 1). There were also 14 refugees (0.9%) with more than one infection. 

**Figure 2:**
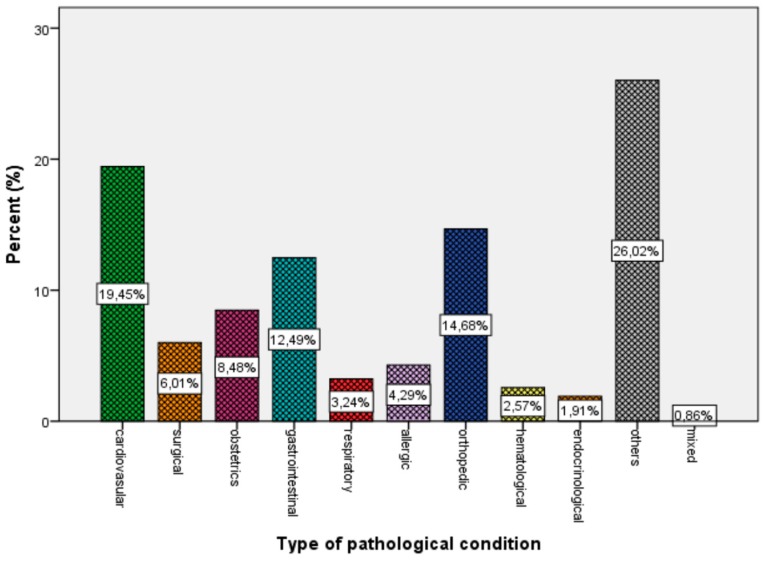
Percentage of system affected in non-infectious diseases for the whole population.

For non-infectious conditions, cardiovascular (19.5%), surgical (6.0%), obstetrics-gynecological (8.5%), gastrointestinal (12.5%), respiratory (3.2%), allergic (4.3%), orthopedic (14.7%), hematological (2.6%), and endocrinological (1.9%) disorders were observed. About 26.2% of patients with non-infectious diseases who were seeking care were examined by doctors for different reasons (e.g., consultation, diet guidance), and 0.9% of them suffered from more than one condition (Figure 2).

### 3.3. Child Morbidity

**Figure 3:**
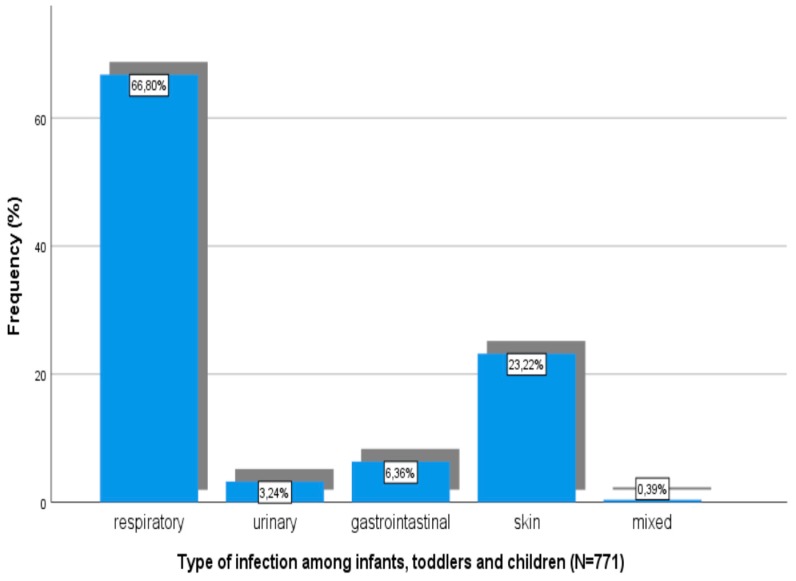
Types of infections among infants, toddlers, and children.

Types of infections among children are shown in Figure 3.

Respiratory track infection was the most common disease in the age groups of infants (48.8%), toddlers (54.4%), and children (58.9%) (Table 2). On the other hand, in the age groups of adolescents (49.8%) and adults (55.3%), the most frequent reason for medical advice or care seeking was a non-infectious or non-communicable condition or disease. 

It is worth mentioning that toddlers were 1.564 times (*p* = 0.027) more likely to get ill comparing to infants (Table 2). 

However, statistical analysis revealed that there was no association between gender and morbidity (*p* = 0.422). (RR: Relative Risk, C.I.: Confidence Interval)

**Figure 4:**
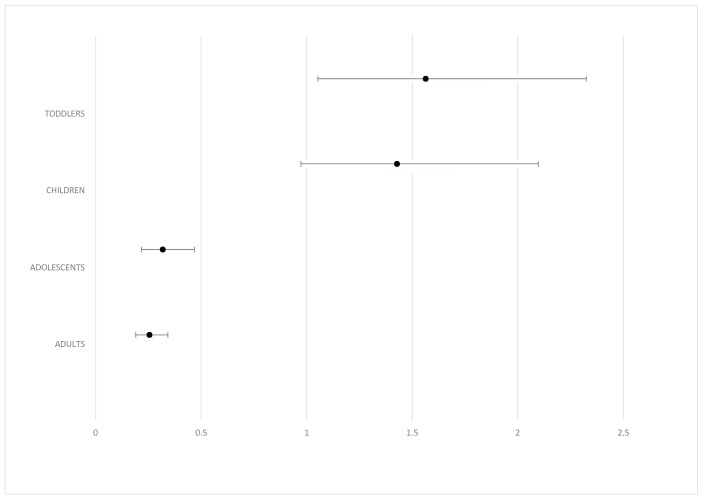
Odds ratio and CI for overall morbidity in comparison to the age group of infants.

Odds ratio and confidence intervals for overall morbidity in comparison to infants are shown on Figure 4. Toddlers were about 1.564 times more likely to get sick than infants, whereas children were 1.4 times more likely to get sick. 

Lastly, only 1.4% of the patients who were examined were taken to hospital for further examination and treatment. 

## 4. Discussion

The main finding of our study is that during a 6-month winter period, most of the children’s visits to the doctor’s office in refugee camps were due to infectious diseases (66,8%). Respiratory tract infections accounted for the majority of communicable diseases, with toddlers being more likely to suffer from one in comparison to the other age groups. Despite this relatively high morbidity due to infectious diseases, the percentage of referrals to tertiary hospitals was significantly low, and no serious cases of vaccine-preventable disease outbreaks and mortality were noted.

This finding comes in accordance with the results of other studies conducted over the last decade, in which infectious diseases are mentioned as the main health problem between asylum seekers [8,9]. In a very recent study that took place in Greece, members of Syrian American Medical Society Global Response (SAMS—GR), who provided medical services in four refugee camps in northern Greece, examined about 7500 people of all ages over a three-month summer period (June 2016–August 2016), most of whom were also adults [10]. Female patients (51.8%) outnumbered the male ones (48.2%). In the aforementioned study, respiratory and gastrointestinal infections were the main reason for care seeking among refugees in Northern Greece.

Analyzing our infection data bank, diseases of respiratory tract were the most common among other conditions. Of all patients, 37.5% were diagnosed with a respiratory infection, while 12.8% had skin infections. Urinary and gastrointestinal infections accounted for only 3.9% and 3.7% of all infectious diseases, respectively, which indicates that overall hygiene conditions in the camps were of a relatively good level. Only 14 patients (0.5%) were diagnosed with more than one infection. Multimorbidity was also negatively associated with immigrants and refugees in another study [11]. Previous studies which took place in Greece and Brussels also reported that respiratory symptoms were the most common finding among the patients [9,10,11,12]. The resettlement of asylum seekers to a different environment and the exposure to other respiratory viruses may explain this fact [13]. Similar findings (especially respiratory, digestive, and skin conditions among newly arrived refugees) were also reported in other studies [9,10,11,12]. A possible explanation for the prevalence of these diseases might be the poor state of migrants’ health and the questionable hygienic conditions during their travel [8,12,14]. Overcrowded camps and poor living conditions may have made migrants more vulnerable to communicable illnesses as well, especially from the respiratory and gastrointestinal system [8,9,10,11,12,13,14,15]. In their study, Bloch-Infanger et al. claim that the percentage of the migrant population seeking for medical care has increased among young people during the last decade [8]. Furthermore, these patients were most frequently referred and hospitalized [8]. Despite the fact that communicable diseases were the most common reason for care seeking in the population of our study, overall referrals to tertiary hospitals were very few. This may be an indicator of adequate first level medical care and easy access to the doctor’s office. Appropriate access to medical care regardless of the legal status of each country is vital in order to improve health status and enhance the prevention of contagious diseases [16].

Regarding non-infectious conditions (41.62%), these were classified in eleven categories based on system affected: cardiovascular (19.45%), surgical (6.01%), obstetrics-gynecological (8.48%), gastrointestinal (12.49%), respiratory (3.2%), allergic (4.29%), orthopedic (14.68%), hematological (2.57%), and endocrinological (1.91%). Approximately 26.02% of patients were examined by doctors for different reasons and 0.86% had more than one pathological issue. Some studies indicate that chronic diseases and psychiatric conditions are the most important health problems among migrants and refugees hosted in camps [17,18]. In our study, neither chronic disorders, such as Hepatitis A, B, or C infections and tuberculosis, nor mental issues, such as depression, were observed. Lastly, no patients with dental problems sought medical care, in contrast to van Berlaer et al., who reported 10% of refugees had dental issues and visited the doctor’ s office [9], and a study which took place at P. and A. Kyriakou Children’s Hospital, a tertiary pediatric hospital in Athens, Greece, which showed that between immigrant children who were examined clinically at the hospital, dental abnormalities (21%) were the most frequent medical problem identified. In our study, other disorders, such as respiratory and dermatological infections, genitourinary, cardiological, and surgical matters requiring further intervention, existed in 7.3% of this research population. [19]. We have to clarify that long-term conditions (including vector-borne and blood-borne infections, such as malaria, leishmaniasis, and *Helicobacter Pylori*) may have been underrepresented or under-diagnosed, as health care providers focused on identifying acute conditions.

In our study, we also compared the disease burden among different age groups. This comparison revealed that respiratory infections are the most common disease in the age groups of infants (48.8%), toddlers (54.4%), and children (58.9%), whereas in the age groups of adolescents (49.8%) and adults (55.3%), the most frequent disorder is non-infectious. Pavli et al. also reported that refugee children are prone to respiratory and gastrointestinal issues, as well as skin conditions [12]. In our study, we examined the relative chance for infection between age groups and we found out that toddlers were 1.564 times (*p* = 0.027) and children were 1.428 times (*p* = 0.069) more likely to suffer from communicable diseases compared to infants (Figure 4). This comes in accordance with other studies as well. For instance, van Berlaer et al. reported that almost two-thirds of children younger than 5 years of age suffered from diseases [9]. Lastly, our study shows that there is no association whatsoever between gender and likelihood of someone getting sick. Even though some studies report female immigrants being more vulnerable to health disorders, in our study no such association was noted [12]. 

To our knowledge, this is the first study examining disease burden and child morbidity in Greek refugee camps during a 6-month winter period. Refugee camps in mainland Greece were established in a very short period of time with few resources, mainly as temporary dwellings that were meant to accommodate people for short-term stays only. However, the closure of the “Balkan route” and the EU-Turkey agreement has lead Greek refugee camps to become overcrowded long-term shelters overnight. This new refugee sheltering model, along with the fact that conflicts in Syria are still in progress, has prompted the need to have its functionality and effectiveness assessed, especially in terms of medical care and morbidity. Thus, our study fills a gap in the existing literature shedding, light on the overall disease burden during the winter period and examining how child groups shoulder this burden. 

A number of limitations exist. Even though we excluded from our database double reports for the same patient and condition, there were patients visiting the primary care office two or three times, thus not allowing for the overall risk to be calculated. Additionally, we focused on the group of children less than 12 years old and excluded adolescents (who in terms of infectious diseases demonstrate more common morbidity to adults) from our analysis. A study that could compare refugee morbidity to that of the local population would provide better understanding of the overall disease burden and guide possible prevention interventions.

In conclusion, communicable diseases are the most common cause for care seeking among refugees in camps in mainland Greece during a winter period, with children and especially toddlers suffering more often from respiratory tract illnesses than other age groups. This urgently-established refugee hosting model in Greece seems to be working effectively in terms of primary medical care and hygiene levels.

## Figures and Tables

**Table 1:** Demographic Data.

Age Group	N (%)
Infants	258 (9.8)
Toddlers	333 (12.7)
Children	353 (13.4)
Adolescents	209 (7.9)
Adults	1453 (55.2)
**Total**	**2631 (100)**

**Table 2:** Relative risk of suffering from an infectious disease among age groups.

	*p*	RR	95% C.I.	
			Lower	Upper
Toddlers vs. Infants_	0.027	1,564	1,053	2,325
Children vs. Infants	0.069	1,428	973	2,097
